# Supplementary figures and images for: Early Warning Signs in Social-Ecological Networks
Source: PLoS One. 2014 Jul 11;9(7):e101851. doi: 10.1371/journal.pone.0101851 (PMC4094384; doi:10.1371/journal.pone.0101851)

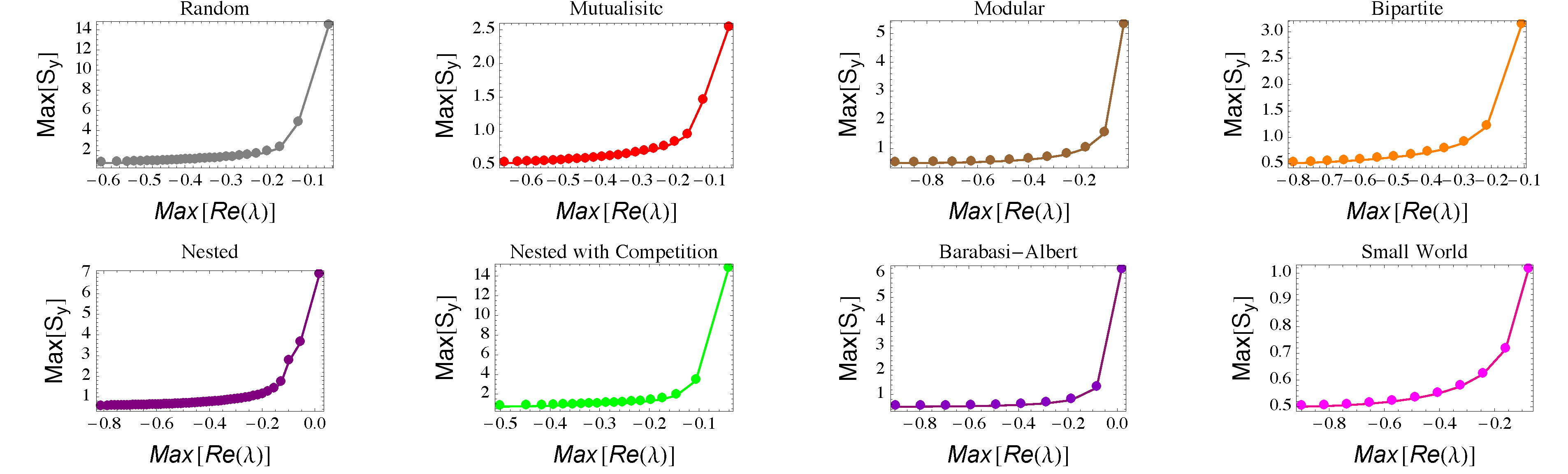

Supplement: Figure S1 — Increase in Max [Sy] as Max [Re(λ)] tends to zero for mean field networks of size, N = 20, C = 0.2. Increasing values of Max[Re(λ)] are obtained by increasing the interaction strength, p. The plotted values are the ensemble averages of 1000 realizations. (TIFF) [file pone.0101851.s001.tif]

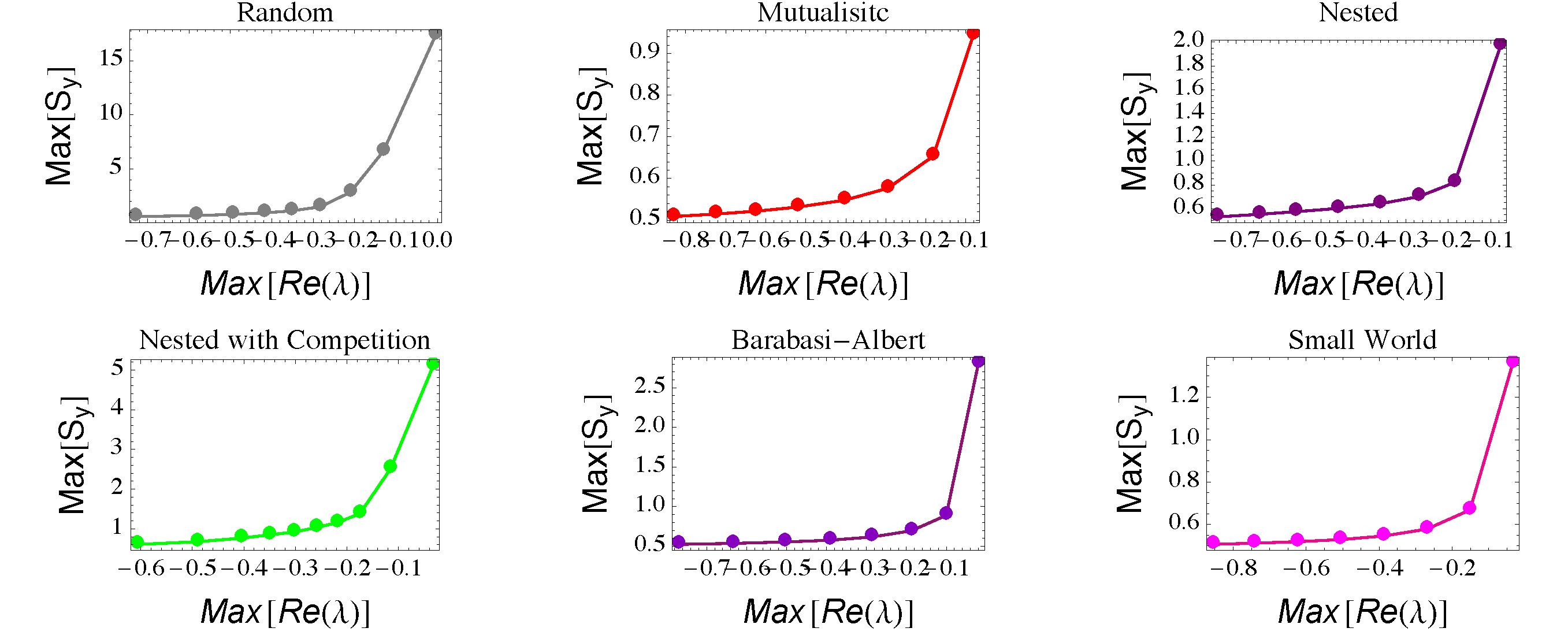

Supplement: Figure S2 — Increase in Max [Sy] as Max [Re(λ)]→0 for mean field networks of size, N = 20, p<<pc. Increasing values of Max[Re(λ)] are obtained by increasing the connectivity, C. The plotted values are the ensemble averages of 1000 realizations. (TIFF) [file pone.0101851.s002.tif]

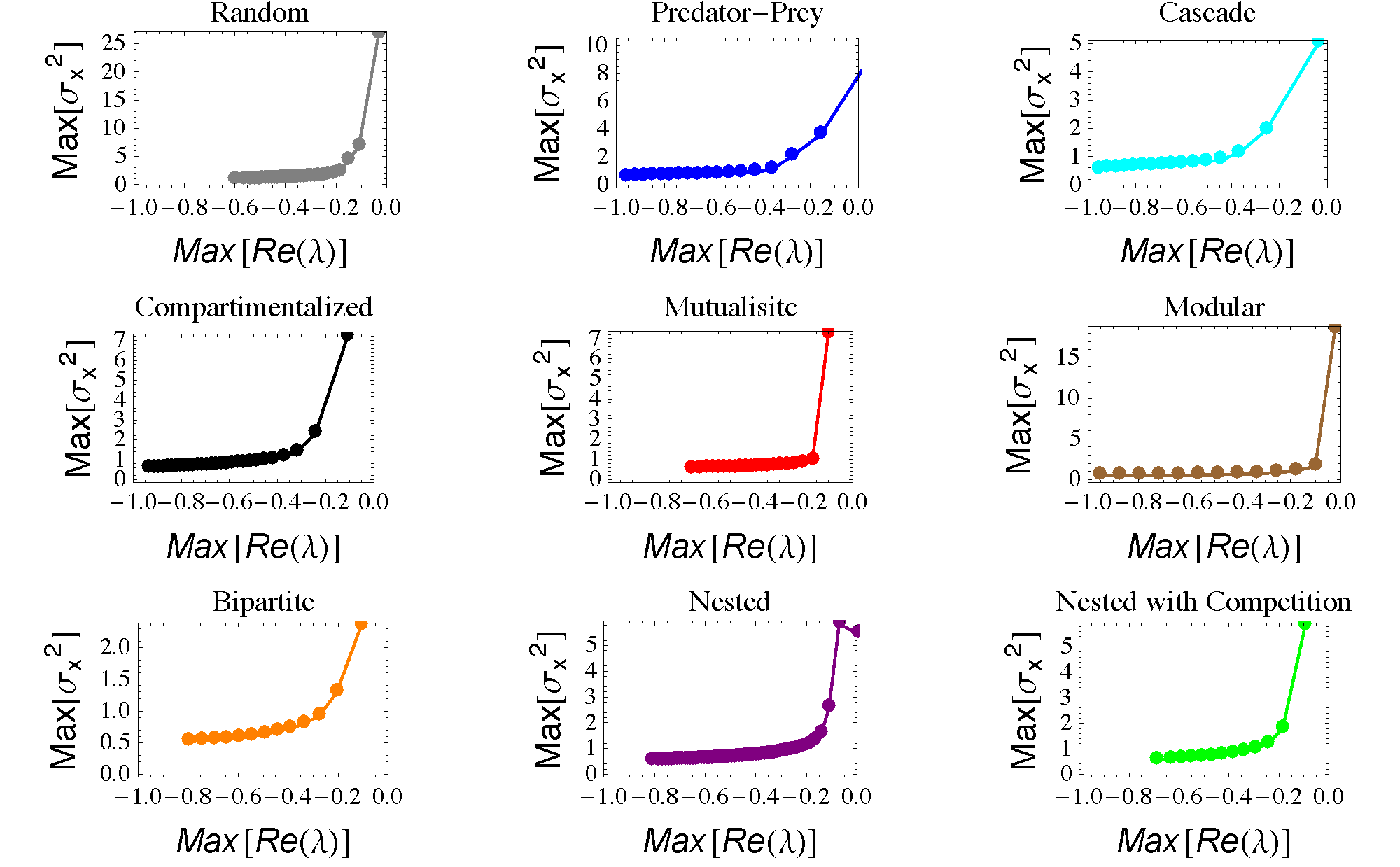

Supplement: Figure S3 — Increase in Max [Sy] as Max [Re(λ)] tends to zoro for complex networks with “weak” disorder (see Section 1) of size, N = 20 and C = 0.2. Increasing values of Max[Re(λ)] are obtained by increasing the interaction strength, p. The plotted values are the ensemble averages of 1000 realizations. (TIFF) [file pone.0101851.s003.tif]

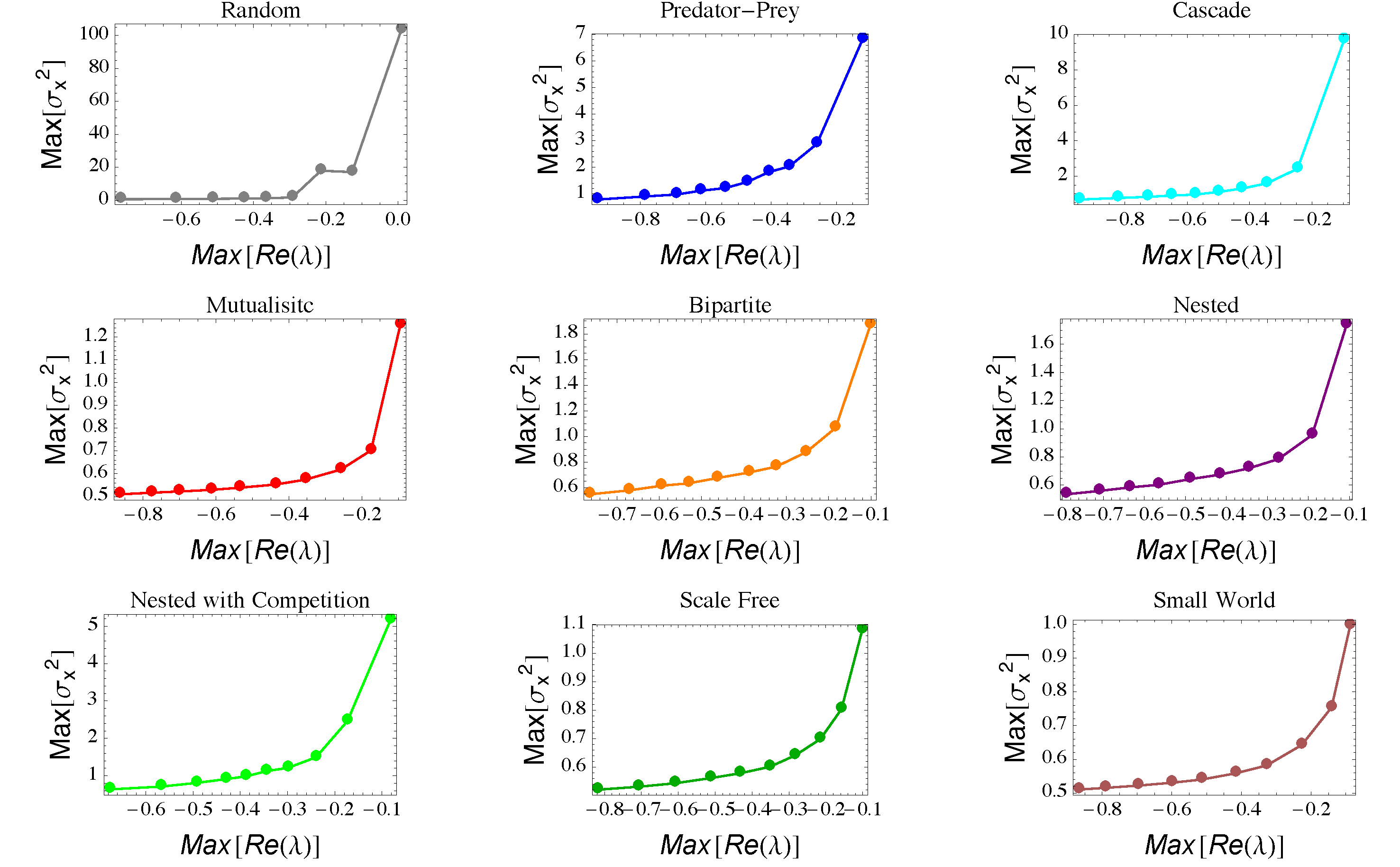

Supplement: Figure S4 — Increase in Max [Sy] as Max [Re(λ)]→0 for complex networks with “weak” disorder (see Section 1) of size, N = 20 and p<<pc. Increasing values of Max[Re(λ)] are obtained by increasing the connectivity, C. The plotted values are the ensemble averages of 1000 realizations. (TIFF) [file pone.0101851.s004.tif]

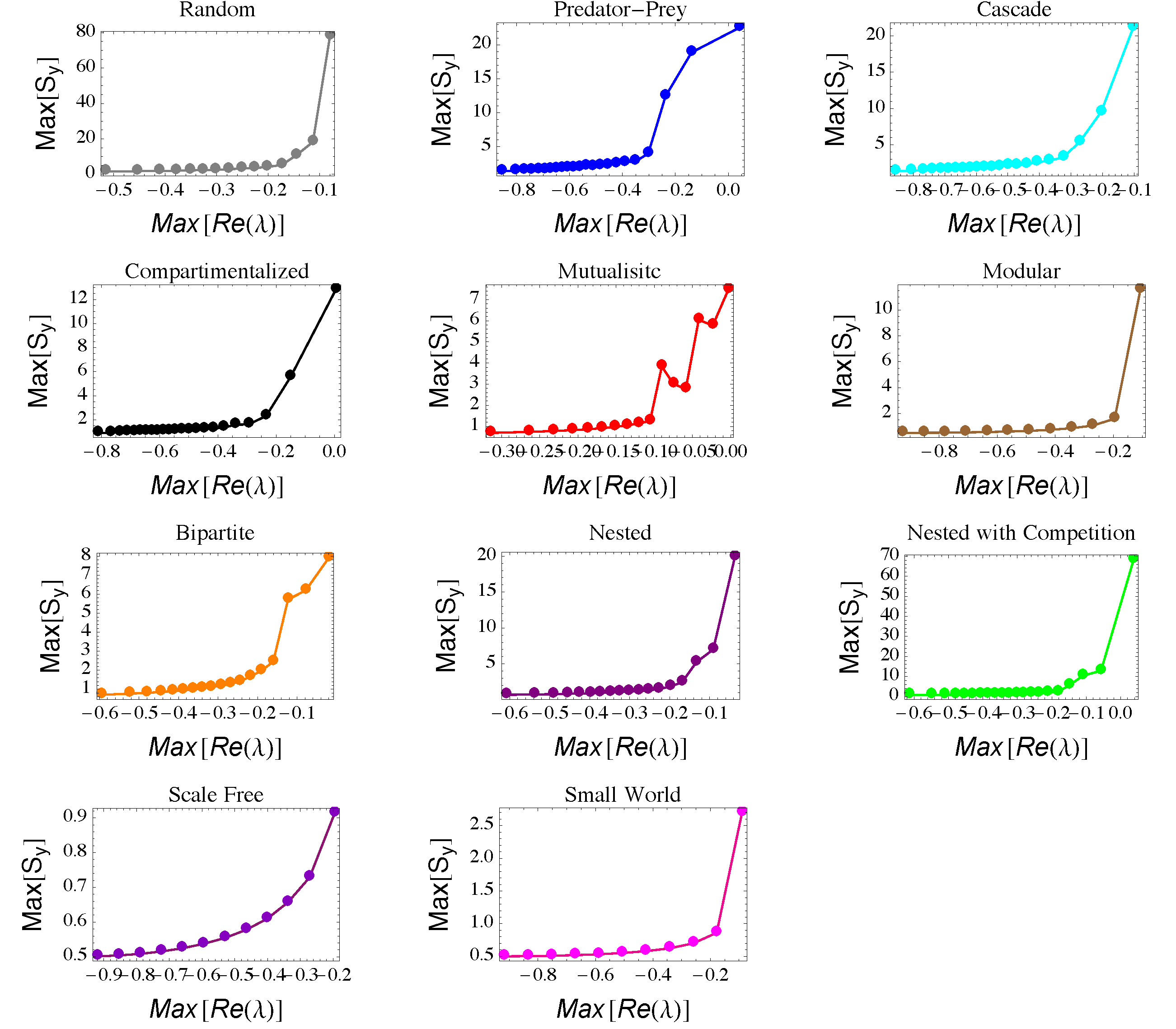

Supplement: Figure S5 — Increase in Max [Sy] as Max [Re(λ)] tends to zero for complex networks with “strong” disorder (see Section 1) of size, N = 20 and C = 0.2. Increasing values of Max[Re(λ)] are obtained by increasing the interaction strength, p. The plotted values are the ensemble averages of 1000 realizations. (TIFF) [file pone.0101851.s005.tif]

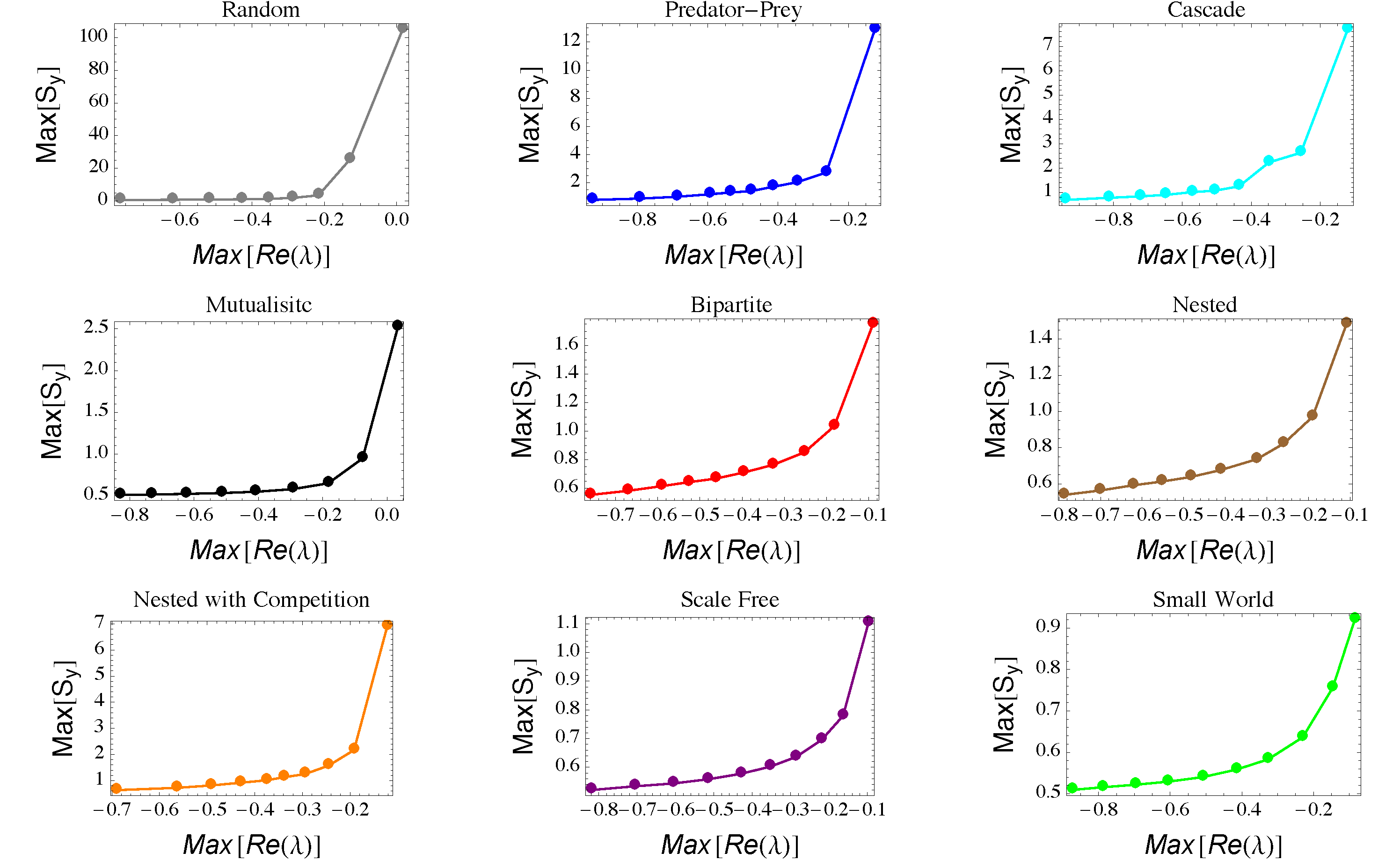

Supplement: Figure S6 — Increase in Max [Sy] as Max [Re(λ)] tends to zero for complex networks with “strong” disorder (see Section 1) of size, N = 20. Increasing values of Max[Re(λ)] are obtained by increasing the connectivity, C. The plotted values are the ensemble averages of 1000 realizations. (TIFF) [file pone.0101851.s006.tif]

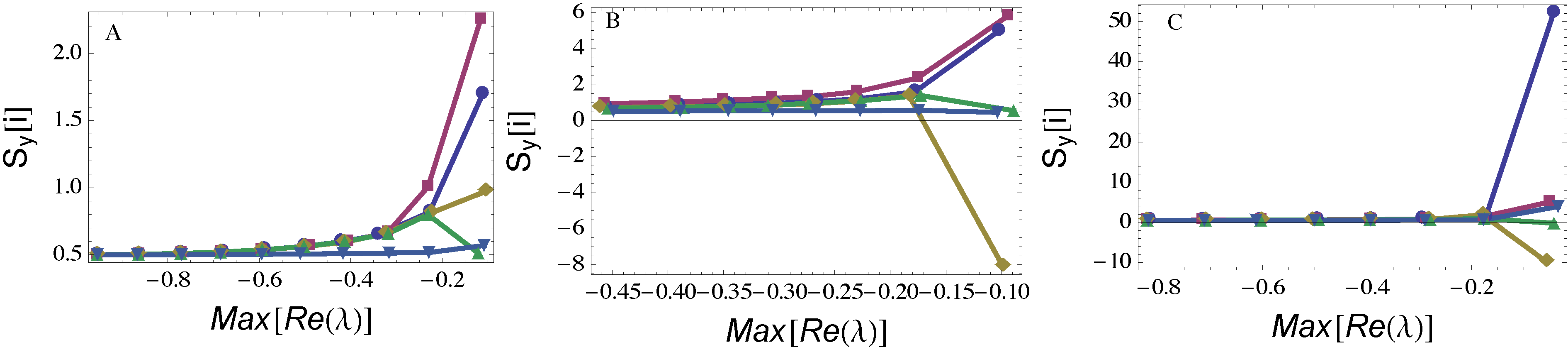

Supplement: Figure S7 — Elements of the covariance matrix Sy corresponding to nodes with the highest number of connections (green), lowest number of connections (light blue), highest eigenvector centrality (gold), max[Sy] (violet) and max[Sy]-min[Sy] (purple), in the case of: (A) mutualistic, (B) mutualistic nested with competition, (C) small world interactions, for mean field networks (of size N = 20 and connectivity C = 0.3). The plotted values are the ensemble averages of 1000 realizations. (TIFF) [file pone.0101851.s007.tif]

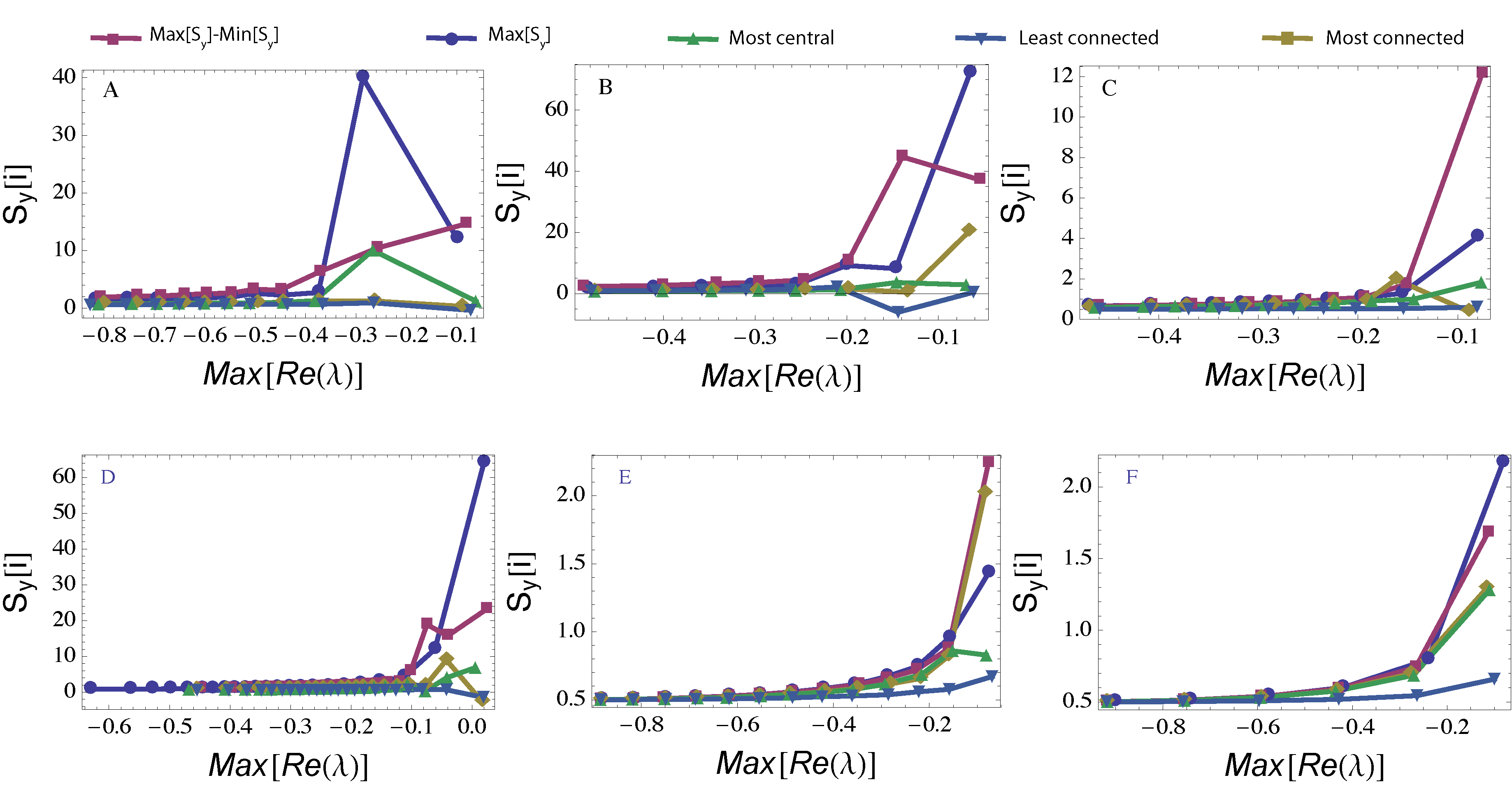

Supplement: Figure S8 — Elements of the covariance matrix Sy corresponding to nodes with the highest number of connections (green), lowest number of connections (light blue), highest eigenvector centrality (gold), max[Sy] (violet) and max[Sy]-min[Sy] (purple) in the case of (A) random, (B) predator-prey, (C) mutualistic, (D) mutualistic nested with competition, (E) Small world, (F) Barabasi-Albert, networks with strong disorder (of size N = 20 and connectivity C = 0.3). The plotted values are the ensemble averages of 1000 realizations. (TIFF) [file pone.0101851.s008.tif]

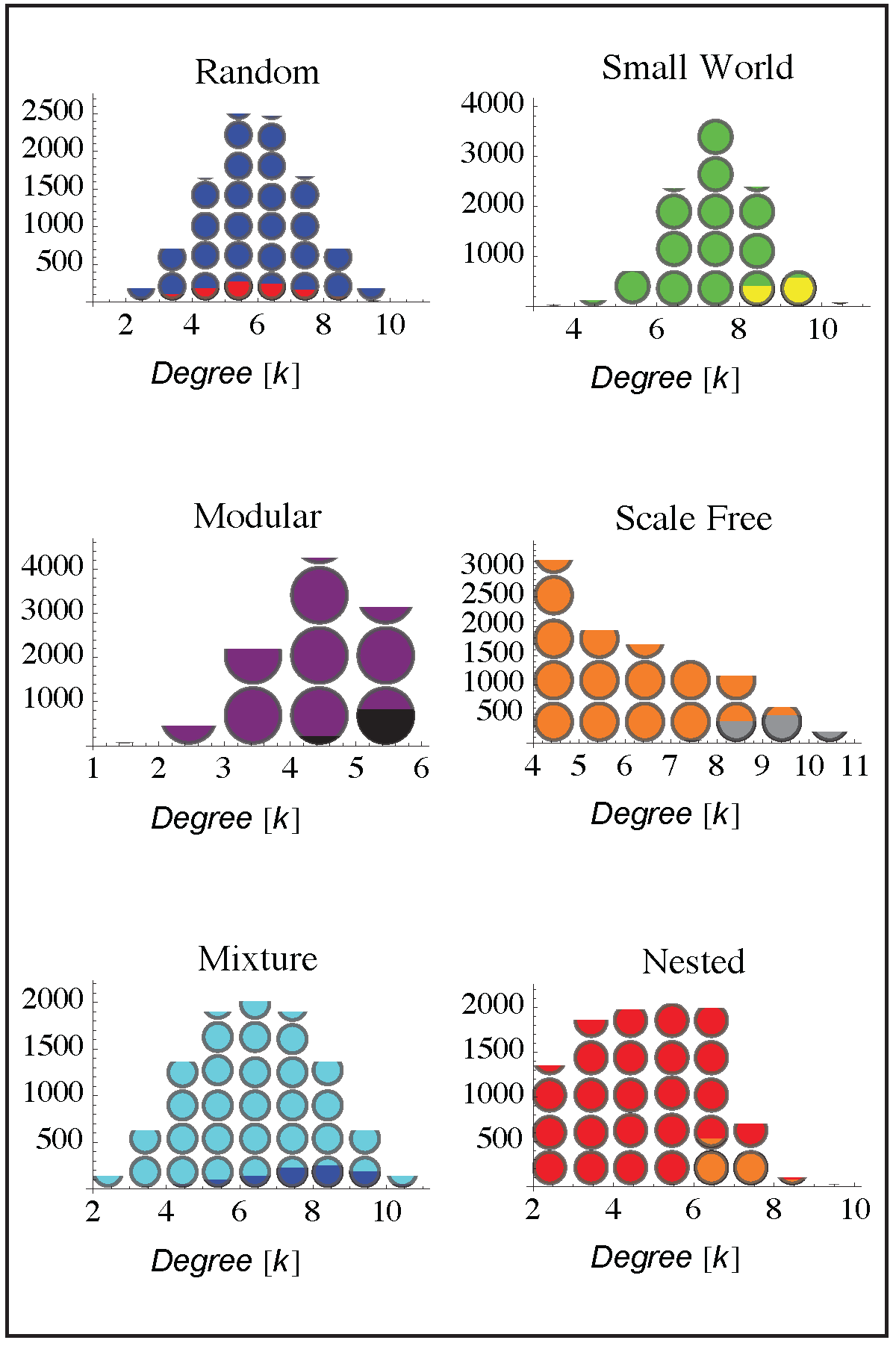

Supplement: Figure S9 — Frequency distribution of the degrees (i.e., number of connections) of the networks' nodes and (with partially filled circles) of the nodes associated with the maximum value of the covariance matrix Sy in mean field networks with a variety of interactions. Based on a set of 100 realizations. Notice how, in mutualistic networks the node corresponding to max[Sy] is associated with the nodes with the highest degrees (i.e. the generalist species). (TIFF) [file pone.0101851.s009.tif]

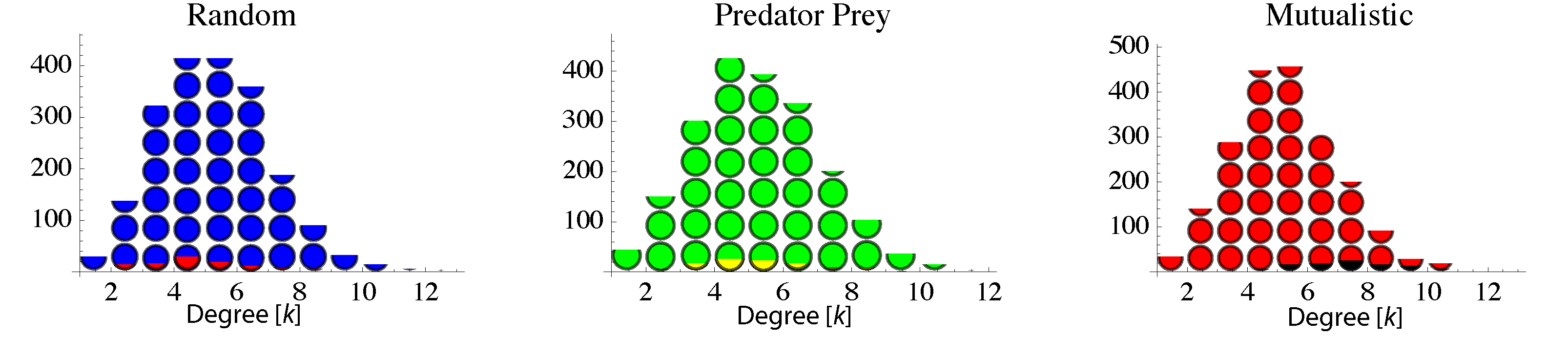

Supplement: Figure S10 — Frequency distribution of the degrees (i.e., number of connections) of the networks' nodes, and (with partially filled circles) frequency distribution of the nodes associated with the maximum value of the covariance matrix Sy in “strongly” disorganized networks with a variety of interactions. Based on a set of 100 realizations. Notice how, in mutualistic networks the node corresponding to max[Sy] is associated with the nodes with the highest degrees (i.e. the generalist species). (TIFF) [file pone.0101851.s010.tif]

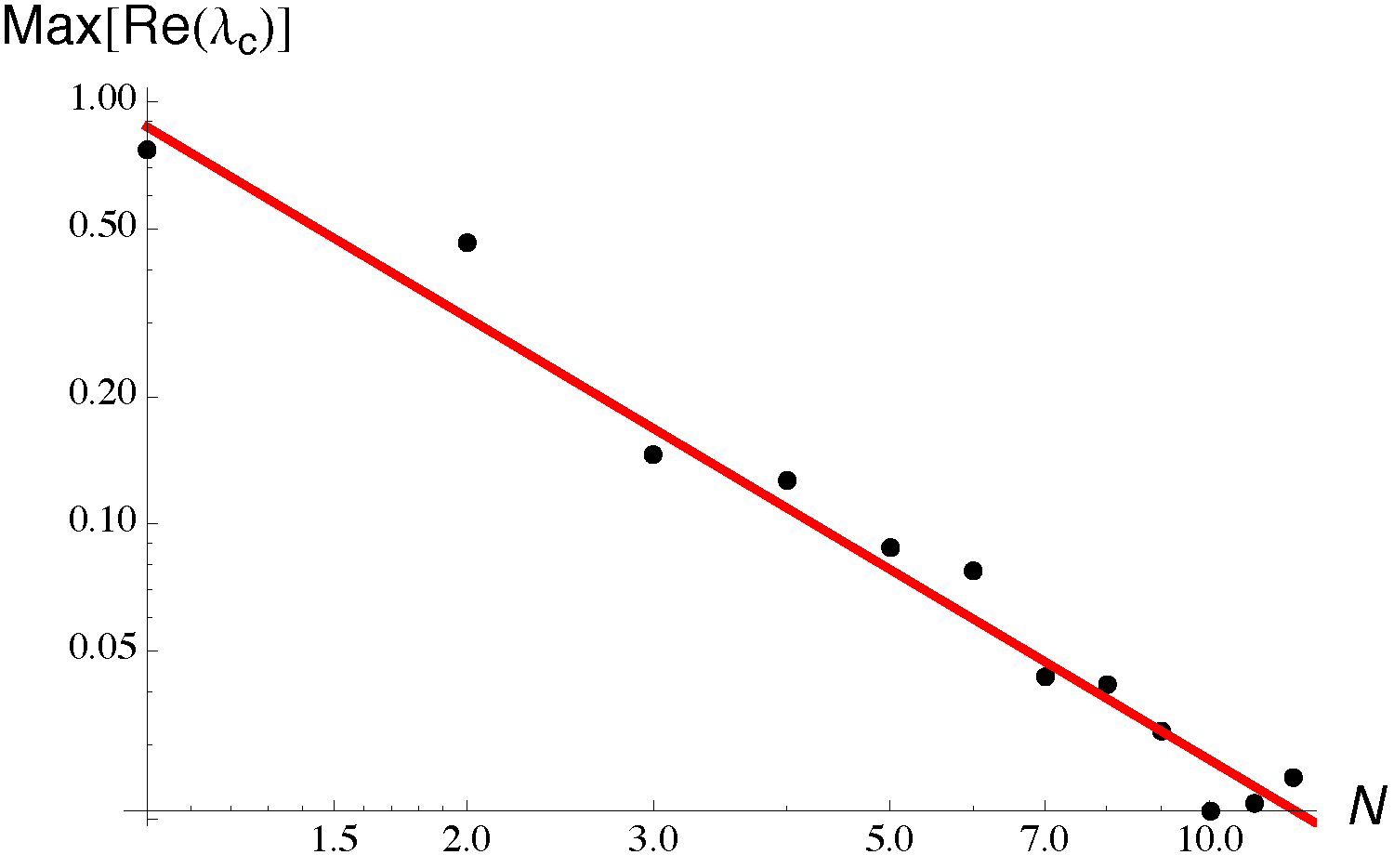

Supplement: Figure S11 — Effect of the network size on the magnitude of the early warning sign. Maximum real part (in absolute value) of the network's eigenvalues as a function of the network size, N for a random network with C = 0.25 and p = p c = 1/√NC. As N increases the max of Re(λ) tends to zero as Max[Re(λ)] ∼N−1.5 and the resilience of the system decreases, while the “height” of the early warning increases. Therefore, as N increases, the early warning sign becomes sharper (see also Figure 1 in the main text). (TIFF) [file pone.0101851.s011.tif]

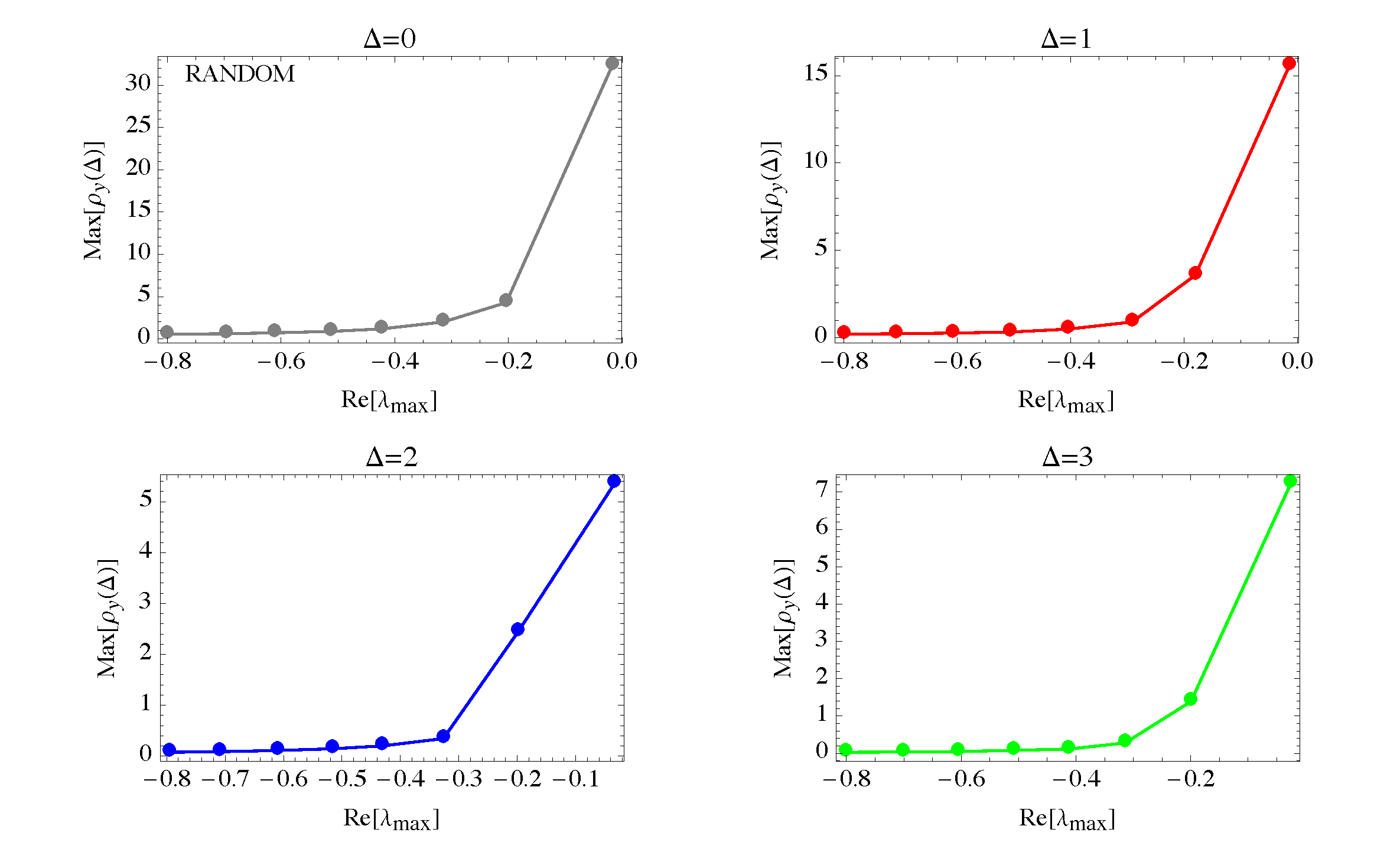

Supplement: Figure S12 — Increase in Max [ρy] as Max [Re(λ)] tends to zero for “strongly” disordered networks with a random architecture with N = 20 and C = 0.3. Increasing values of Max[Re(λ)] are obtained by increasing the interaction strength, p. The plotted values are ensemble averages of 100 realizations. (TIFF) [file pone.0101851.s012.tif]

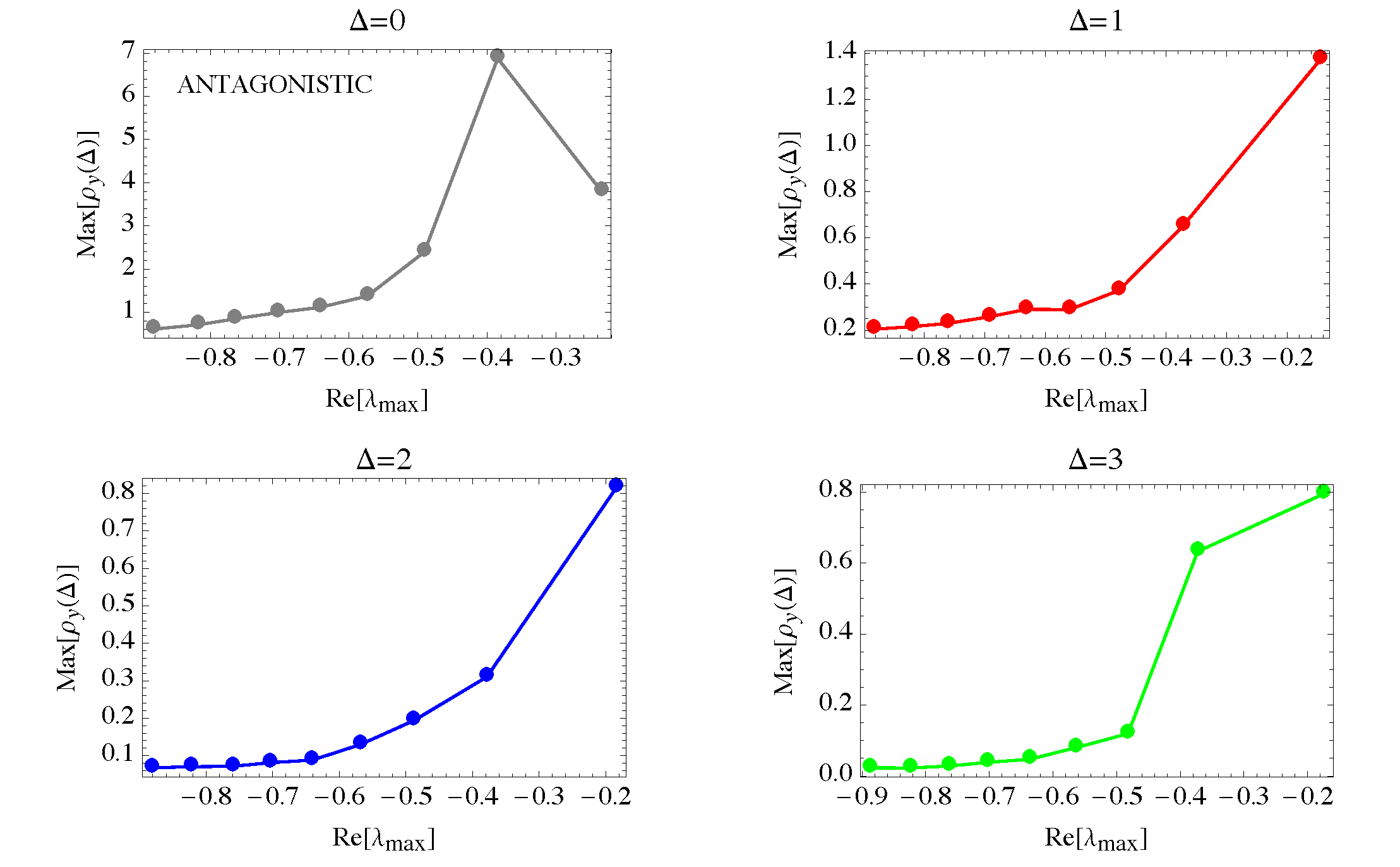

Supplement: Figure S13 — Increase in Max [ρy] as Max [Re(λ)] tends to zero for “strongly” disordered networks with a predator-prey architecture and N = 20, C = 0.3. Increasing values of Max[Re(λ)] are obtained by increasing the interaction strength, p. The plotted values are ensemble averages of 100 realizations. (TIFF) [file pone.0101851.s013.tif]

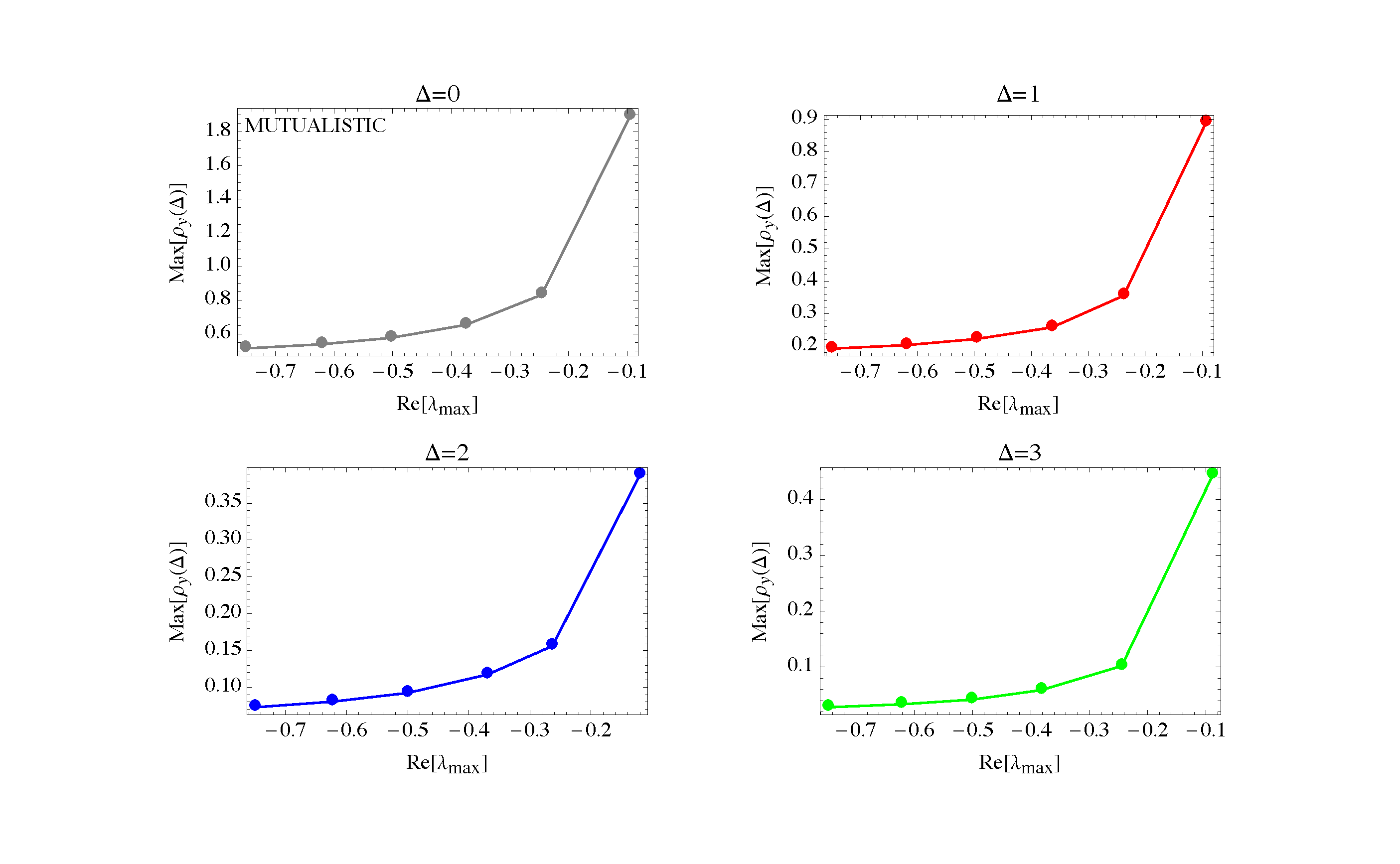

Supplement: Figure S14 — Increase in Max [ρy] as Max [Re(λ)] tends to zero for “strongly” disordered networks with a mutualistic architecture and N = 20, C = 0.3. Increasing values of Max[Re(λ)] are obtained by increasing the interaction strength, p. The plotted values are ensemble averages of 100 realizations. (TIFF) [file pone.0101851.s014.tif]

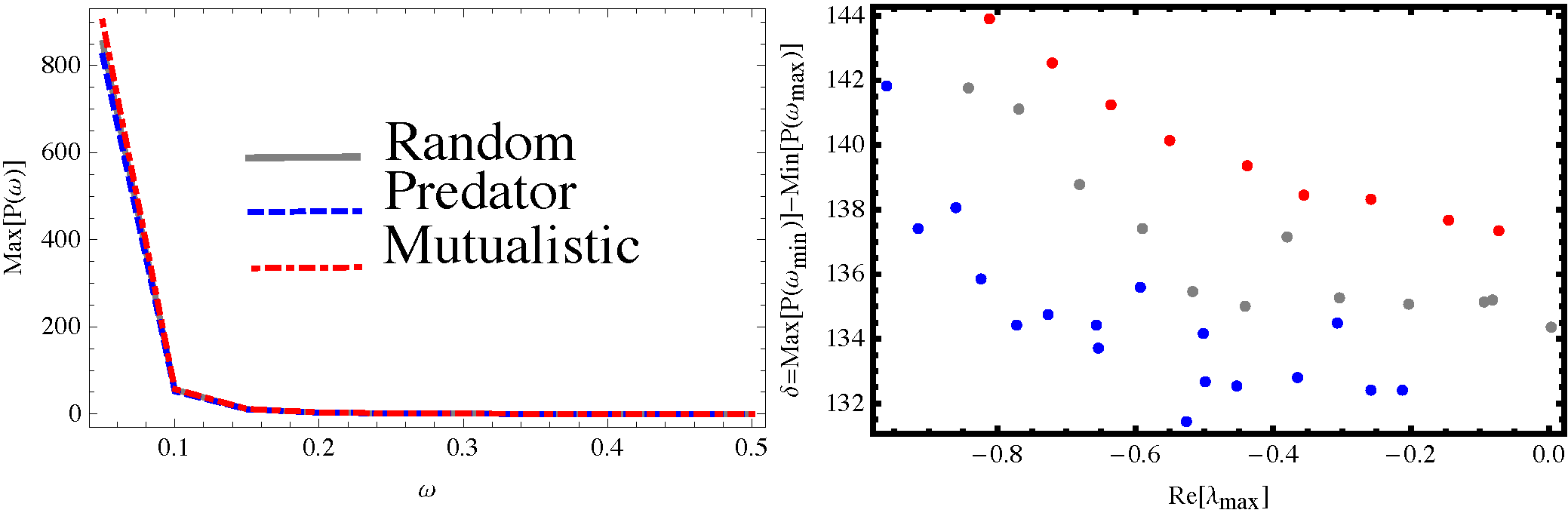

Supplement: Figure S15 — Left panel: max element of the power spectrum matrix as a function of frequency for three different architectures. The impact of the structure on the spectrum is negligible. Right panel: Power spectrum evaluated in the minimum and maximum frequency as p tends to pc (and thus Max[Re(λ)] tends to zero) for strongly disordered systems (N = 20 and C = 0.2) with random, predator-prey, and mutualistic interactions. Increasing values of Max[Re(λ)] lead to a decrease in δ = Max[P(ωmin)] - Max[P(ωmax)], that therefore might be considered a precursor for a critical transition. However, the intensity of this early warning sign is quite weak and thus difficult to detect. The plotted values are ensemble averages of 100 realizations. (TIFF) [file pone.0101851.s015.tif]

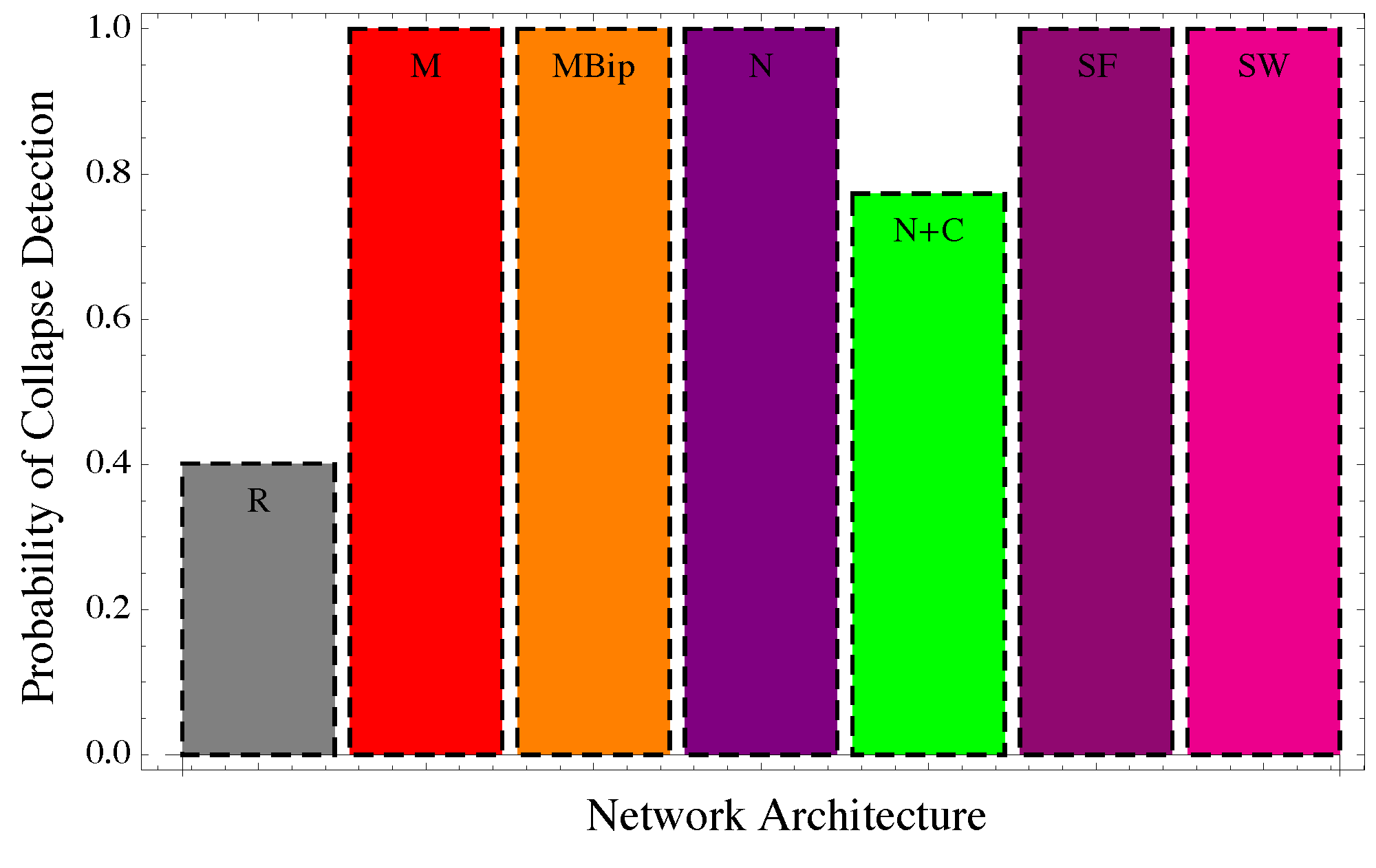

Supplement: Figure S16 — Probability of detecting true positives (i.e. of not missing a warning sign) in the case of mean field networks, using the same detection criteria as in Figure 4 . (TIFF) [file pone.0101851.s016.tif]

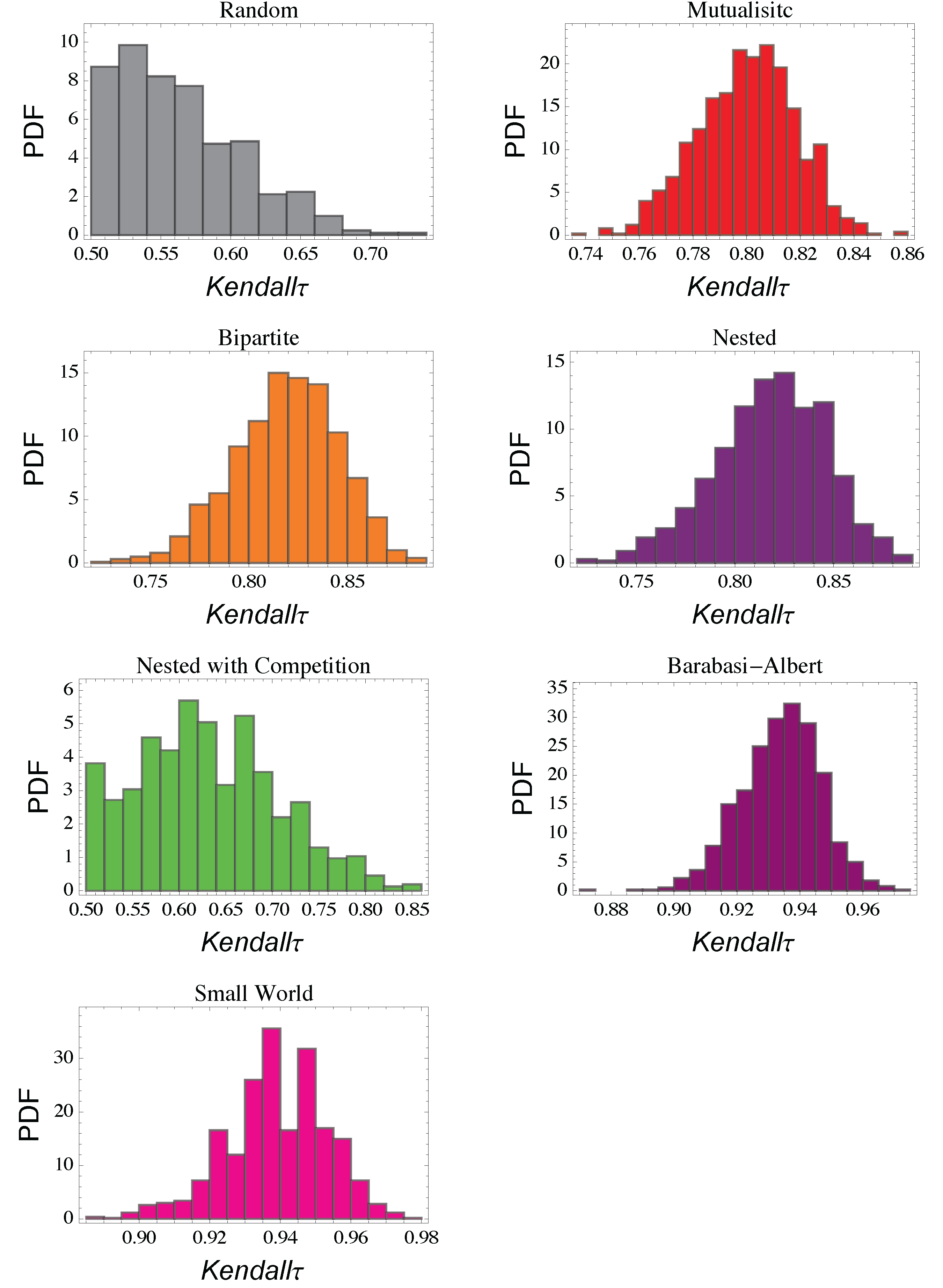

Supplement: Figure S17 — Frequency distribution of the ρK statictics used to detect early warning signs of instability in the case of mean field networks. (TIFF) [file pone.0101851.s017.tif]

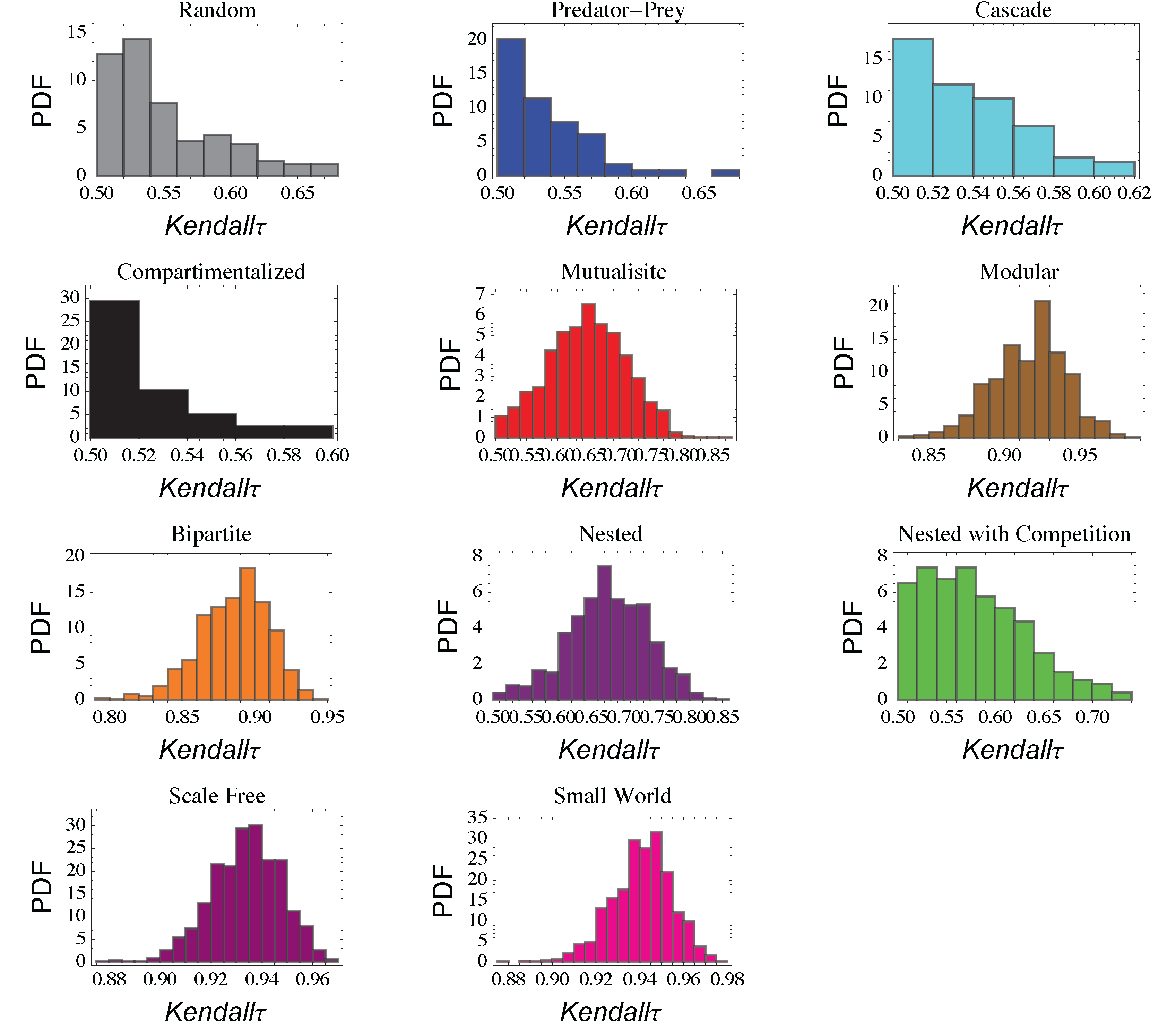

Supplement: Figure S18 — Frequency distribution of the ρK statictics used to detect early warning signs of instability in the case of full disordered networks. (TIFF) [file pone.0101851.s018.tif]
