# Supplementary material for: Early Warning Signs in Social-Ecological Networks
Source: PLoS One. 2014 Jul 11;9(7):e101851. doi: 10.1371/journal.pone.0101851 (PMC4094384; doi:10.1371/journal.pone.0101851)
Supplement: Materials S1 — (DOC) [file pone.0101851.s019.doc]

**Supplementary online materials**

**S1. Structures of Socio-Ecological Interacting systems.**

a) *Random matrix* [1] with connectivity *C.* We pick an element A_i,j_ at random, and with probability *C* we assign a link between nodes *i-j* and *j-i* (A_j,i_). Each of these two links has a weight (or strength), *p*, that is positive (or negative) with probability 0.5; b) *Antagonistic matrix* [2], where connections are again random (with probability *C*), but if A_i,j_ has sign +(–), then A_j,i_ has sign – (+); c) *Cascade network* [3]: links occur with a given probability, but form a hierarchical structure, whereby there is a top “predator” (with sign +) that feeds on all the other species (with sign -); then there is the second top predator, and so on; species left with the lowest ranking function as producers; d) *Compartmentalized* structure [4], formed by groups of antagonistic species that interact only with species within their own group; e) *Mutualistic network* (6), with a random matrix, but where both A_i,j_ and A_j,i_ have positive signs (++); f) *Modular mutualistic* matrix [5]: nodes are divided into communities that positively interact within their groups (++). g) *Bipartite Mutualistic* matrix [6]: nodes are divided into two groups, and each node positively interacts only with nodes of the other group (++); h) *Bipartite Nested Mutualistic* [7]: a bipartite graph is generated with hierarchical structure where specialist nodes (i.e., with only few mutualistic links) tend to interact with a suitable subset of the mutualistic partners of the generalist nodes (++); i) *Nested Mutualistic with Competition* [8]: nodes are divided into two groups; each node positively interact with nodes of the opposite group (++), while compete with nodes of the same group (--); j) *Barabasi-Albert (BA) networks* [9]: binary one-zero networks are generated with power-law degree distribution. This algorithm simulates a preferential attachment process, in which a new vertex with *d* edges is added at each step. The BA graph displays a scale-free behavior that strongly correlates with the network's robustness to failure. We then assign to each link a positive weight *p*; The connectivity of the BA networks is controlled indirectly by the parameter *d*. In our simulation we set *d*=1 to generate low connectivity networks, *d*=2 for average connectivity and *d=*4 for high connectivity. k) *Watts-Strogatz (WS) network* [10]: this is a one-parameter (degree of disorder r) model that interpolates between an ordered finite dimensional lattice and a random graph. Its main property is to display both high clustering coefficient and small world property. The WS model displays this duality for a wide range of the rewiring probabilities r. In our simulation we used r=0.3. The connectivity of the WS model depends on the starting 2k-regular graph [10]. For low connectivity we set k=2; for average connectivity k=4; for high connectivity k>5.

The linkage density in each network is described by the parameter *C* (the connectivity ).

We generate each of the structures (*a*)-(*k*) keeping constant the connectivity *C* and changing the strength of the interactions (alternatively, we fix *p* and vary *C*), until the dynamics become unstable [1,6]. Thus, we use the parameter, *p*, as an indicator of the weights of the interactions between connected nodes (connectivity). We consider three different levels of disorder in the control parameter *p*: 1) *Mean Field:* where we assign a constant value, *p*. We do not consider antagonistic mean field networks (i.e., with constant |*p*| but (+-) interactions) because they are always stable (see below); 2) *Weak disorder*: the interaction strength is drawn from a normal distribution with mean *p* and standard deviation 0.1 *p* (*i.e.,*  ***N***(*p*, *0.1 p*); 3) *Strong disorder*: interaction strengths are drawn at random from ±|***N*** (*0*, *p*)| [6]. Therefore, in all these cases, by increasing the value of *p* (*C*) the resilience decreases until the system become unstable for  *p= p_c_* , or *C=C_c_* [6].

**S2.1 Early Warnings in Multidimensional Mean Field Networks**

In the mean field case with (++) interactions, the matrix is symmetric (**A**=**A**^T^) and therefore all eigenvalues are real numbers. In this case we note that, if **U** is the matrix of the eigenvectors of **A** (i.e. **A** **u**_i_= λ_i_ **u**_i_), then **U** **A U**^T^=**diag**(**λ**), where **diag**( **λ**) is a diagonal matrix with all the eigenvalues of **A**. Therefore in this case we can write Eq. (2) in the main text as:

$\mathbf{U} \mathbf{AU}^{\mathbf{T}}\mathbf{U} \mathbf{S}_{\mathbf{y}} \mathbf{U}^{\mathbf{T}}+\mathbf{U} \mathbf{S}_{\mathbf{y}} \mathbf{U}^{\mathbf{T}}\mathbf{U} \mathbf{A} \mathbf{U}^{\mathbf{T}}=-\nu\mathbf{I}= \mathbf{diag} (\lambda) \mathbf{\Sigma}+\mathbf{\Sigma} \mathbf{diag}(\lambda)$,

where $\mathbf{U} \mathbf{U}^{\mathbf{T}}=\mathbf{I}$ and $\mathbf{\Sigma}=\mathbf{U} \mathbf{S}_{\mathbf{y}} \mathbf{U}^{\mathbf{T}}$is a diagonal matrix whose *i*-th eigenvalue is$\zeta_{i}=-\frac{\nu}{2 \lambda_{i}}$. Therefore if the socio-ecological system reaches an instability (i.e. λ_i_=0) for a given *i,* then there is at least one element of the matrix **Σ **and thus of **S_y_** that diverges.

We also notice that in the mean field case we omit the analysis of early-warning performed on antagonistic (+-) matrices, **A’**. In fact, if **A’** is anti-symmetric (i.e., **A’**=-**A’**^T^, and thus with predator prey, cascade or compartmentalized interactions), then the early warning analysis on those matrices is trivial as their stability does not depend on *p* or *C*. In fact, if we multiply the off-diagonal terms of those matrices by the imaginary unit *i*, i.e. **B**=*i* (**A**’-**I A’)**, then **B** is Hermitian and has all real eigenvalues. Therefore **A**’-**I A’** has all pure complex eigenvalues independently of *p* and *C*, which means that **A’** is always stable – given that the self interaction terms are negative constant, i.e. A’_i,i_=-d<0.

Figures S1 and S2 show a rise in *Max*[**S_y_**] as the system approaches instability in mean field networks.

Figure S1

Figure S2

**S2.2 Early Warnings in Complex Networks with Weak Disorder**

Figure S3

Figure S4

**S2.3 Early Warnings in Complex Networks with Strong Disorder**

Figure S5

Figure S6

**S3. Indicators and Node Properties**

In this section we show more analysis on the effectiveness of different node variances as precursors of instability.

Figure S7

Figure S8

Figure S9

Figure S10

Figure S11

**S4. Early Warning for Time Correlation Matrix and Power Spectrum**

We calculate the time lag correlation matrix, **ρ**_y_(Δ) (where Δ is the time lag), and the power spectrum, ***P*** _y_(ω) (where ω is the frequency) for the steady state dynamics:

$\mathbf{\rho}_{\mathbf{y}}(\Delta)=\left\langle\mathbf{y}_{\mathbf{s}}(t),\mathbf{y}_{\mathbf{s}}^{\mathbf{T}}(t+\Delta)\right\rangle=\sigma_{y}(p)Exp(\mathbf{A}(p) \Delta)$ (3)

$\mathbf{P}_{\mathbf{y}}(\omega)=\left|\frac{1}{2\pi}\int_{-\infty}^{+\infty} e^{-i\omega\tau}\rho_{y}(\tau) \text{d}\tau\right|^{2}=\left|-\frac{1}{2\pi}(i\omega-\mathbf{A}(p))^{-1}\nu\mathbf{I}(i\omega+\mathbf{A}^{\mathbf{T}}(p))^{-1}\right|^{2}$ (4)

The following figures show the behavior of the autocorrelation and power spectrum as the system approaches instability.

Figure S12

Figure S13

Figure S14

Figure S15

**S5. Early Warning Detection**

To evaluate whether the onset to instability can be anticipated in time by an increase in *Max*[**S_y_**] (or in other suitably chosen elements of **S_y_**), we test the correlation [11] between *Max*[**S_y_**] and the control parameter (*p* or *C*) that is gradually varied to increase *Max*[Re(λ)] up to a given threshold (here chosen equal to −0.2). If the correlation, ρ_k_, (evaluated with the Kendall-τ test) is significant and greater than 0.5, the increase in *Max*[**S_y_**] is interpreted as an early warning sign. We repeat this analysis for 1000 realizations of the random interaction strength network and determine the distribution of correlations along with the number of realizations with positive warning sign.

Figure S16

Figure S17

Figure S18

**S6. References**

1. May RM (1972) Will a large complex system be stable? Nature 238:413-414.
2. Pimm SL, Lawton JH, Cohen JE (1991) Food web patterns and their consequences. Nature 350(6320): 669-672.
3. Williams R J, Martinez ND (2000) Simple rules yield complex food webs. Nature 404 (6774): 180-183.
4. Stouffer DB, Bascompte J (2011) Compartmentalization increases food-web persistence. Proc. Natnl. Acad. Sci. 108(9): 3648-3652.
5. Newman MEJ (2010) Networks: An Introduction. Oxford University Press.
6. Allesina S, Tang S (2012) Stability criteria for complex ecosystems. Nature 483: 205–208.
7. Bascompte J, Jordano P, Melian C J, Olesen J M (2003). The nested assembly of plant–animal mutualistic networks. Proc. Natl Acad. Sci. USA 100, 9383–9387.
8. Suweis S, Simini F, Banavar J, Maritan A (2013) Emergent structural and dynamical properties of ecological mutualistic networks. Nature. 500:449.
9. Albert R, Barabasi A-L (2002) Statistical mechanics of complex network. Rev. Mod. Phys. 74(1):47.
10. Watts DJ, Strogatz SH (1998) Collective dynamics of ‘small-world’ networks. Nature 393(6684): 440-442.
11. Dakos V, et al. (2012) Methods for detecting early warnings of critical transitions in time series illustrated using simulated ecological data. PLoS ONE 7(7):e41010.
